# Influence of Electrical Heating Metal Mesh and Power Density on Resistance Welding of Carbon Fiber/PEEK Composite

**DOI:** 10.3390/polym14132563

**Published:** 2022-06-23

**Authors:** Donglu Wei, Yizhuo Gu, Hanrui Zhu, Min Li, Shaokai Wang

**Affiliations:** 1 School of Materials Science and Engineering, Beihang University, Beijing 100191, China; mit_ing@163.com (D.W.); zhuhanrui1995@163.com (H.Z.); leemy@buaa.edu.cn (M.L.); wsk@buaa.edu.cn (S.W.); 2 Research Institute for Frontier Science, Beihang University, Beijing 100191, China

**Keywords:** resistance welding, carbon fiber composite, PEEK, electrical heating element

## Abstract

An experimental investigation on the resistance welding of carbon-fiber-reinforced polyetheretherketone (PEEK) composite laminate using three types of stainless steel (SS) meshes with different sizes and electrical resistances as heating elements is reported. The objective of this study is to determine the influence of the metal mesh on the welding process and performance at different power densities ranging from 29 to 82 kW/m^2^. Resistance welding equipment is used to monitor the temperature and displacement along the thickness of the laminate. The results show that the power density determines the welding time and heat concentration. A large power density results in a short welding time, but also increases the temperature gradient at the joining interface (almost 50 °C) and causes an obvious deformation of a contraction of more than 0.1 mm along the thickness of the laminate. A SS mesh with low resistance has a strong welding capability, i.e., a high welding efficiency under low power density. A lap shear strength of approximately 35 MPa can be obtained with the appropriate power density. The shear strength is affected by the bonding between the metal mesh and polymer, the metal mesh load bearing, and the metal mesh size.

## 1. Introduction

The emergence of high-performance thermoplastic composites, such as polyetheretherketone (PEEK), polyetherketoneketone (PEKK), polyetherimide (PEI), and polyphenylene sulfide (PPS) matrix composites, has attracted widespread attention in the aviation, automotive, and marine industries because of the low density, excellent mechanical properties, short processing cycles, fire/smoke resistance, recyclability, and almost unlimited shelf life of these composites [1,2]. In particular, thermoplastic matrix composites, unlike thermoset matrix composites, can be welded using fusion bonding techniques [3], which makes them suitable for high-speed assembly processes. Among the various welding methods, resistance welding is easily implemented in industrial applications because of its short processing time and simple equipment required [4,5]. For example, it has been used to weld glass fiber/PPS J-nose leading edges of the Airbus 340–500/600 and 380 airplanes [6]. During resistance welding, an electrical current is applied to a heating element, which is placed between the two adherends. As current passes through the heating element, its temperature increases because of Joule heating. This causes the adjoining polymer to soften (amorphous polymer) or melt (semi-crystalline polymer). The softening or melting of the polymer is followed by a consolidation step, following which the weld interface is cooled under external pressure. The heating element is an important component in resistance welding. Because it is responsible for generating heat at the joining interface and remains embedded in the interfacial region after the welding operation, it has a significant effect on the welding quality and efficiency.

The two types of electrically conductive materials that are mainly used in resistance welding heating elements are carbon fiber (CF) and metal mesh heating elements [4]. The advantage of CF as a heating element is its compatibility with the reinforcing material in welded composites. However, the anisotropic thermal and electrical properties of unidirectional CFs and fabrics lead to difficulties in controlling the temperature distribution during welding [7,8]. Metal mesh heating elements offer a number of advantages compared with CF heating elements. This has led to many studies on metal heating elements in recent years. A stainless steel (SS) mesh is typically used for manufacturing metal mesh heating elements because it provides a uniform temperature distribution over the welding area and produces welds with good consistency and high bonding strength over a wide processing window [4,9,10,11,12,13,14,15].

PEEK is regarded as the most attractive matrix system in the aerospace industry [6,16,17,18]. PEEK is a semicrystalline thermoplastic with excellent mechanical and chemical resistance properties that are retained to high temperatures. These properties have advantages over those of other high-performance thermoplastics, such as PEI and PPS. In particular, continuous carbon-fiber-reinforced PEEK (CF/PEEK) composites are expected to become competitive aerospace material candidates, such as epoxy resin matrix composite and aluminum alloy. Resistance welding has been demonstrated to be a viable technique for joining CF/PEEK composites [6,15,19,20,21]. Dubé et al. found that resistance-welded CF/PEEK exhibits excellent lap shear static and fatigue strengths [9]. However, PEEK has a high melting point and melt viscosity, which increase the difficulties of welding [22]. The welding process must therefore be carefully designed, including heating element, input current, heating time, applied pressure, and cooling method. In particular, the input current should be appropriately controlled to obtain good weld quality. Yousefpour et al. adopted a ramped-power technique for the resistance welding of CF/PEEK in which the input power level is adjusted in-service based on the temperature at the weld interface [23]. Using an optimized power level, a skin/stringer structure with good mechanical performance was successfully resistance-welded [6].

In addition, the heating element is a key factor in resistance welding. The effects of various types of heating elements and surface treatments on CF/PEEK have been investigated. The heating element types investigated include SS meshes with PEEK, SS meshes with PEI, carbon fiber prepreg, and PEI containing multi-wall carbon nanotubes [9,10,11,12,20]. The surface treatments investigated include solvent cleaning, mechanical roughening, and chemical grafting [15,22]. Compared with the extensive research on these two factors, there are comparatively fewer studies on the effects of the SS mesh heating element geometry and power level in the resistance welding of CF/PEEK composites. The geometry of the metal mesh has a significant impact on the weld properties [5,7,12]. Dubé et al. used SS mesh heating elements with different sizes to weld PEKK and PEI composites and found that the open area fraction and wire diameter of the metal mesh are important factors in determining the bonding strength of the welded joints [12]. Because of the high melting point, controlling resistance welding of CF/PEEK composite is more difficult than that of other thermoplastic composites. It is therefore necessary to study and understand the corresponding effects on CF/PEEK to devise a strategy for optimizing the resistance welding process.

The objective of this study is to investigate the effects of the power level and SS mesh geometry on the resistance welding process and the quality of the CF/PEEK laminate. A welding assembly is established, and various power densities of SS mesh heating elements are used to weld CF/PEEK specimens in lap shear joints. The welding process is analyzed by monitoring the temperature at the welding joint and the displacement along the thickness of the laminate. Furthermore, the welding quality is assessed using the lap shear test, and the fracture surfaces characterized using scanning electron microscopy (SEM). The failure mechanism of various welded composites under lap shear loading is discussed. Improvements in welding efficiency and bonding strength using an appropriate power density and SS mesh are demonstrated.

## 2. Materials and Methods

### 2.1. Materials

**Figure A1:**
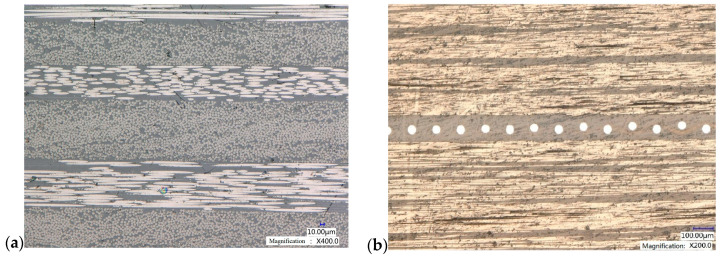
Cross-section image of (**a**) the laminate and (**b**) the welded composite.

The adherends used in this study were supplied by Jiangsu Junhua Ltd., Jiangsu, China and composed of 18 plies of unidirectional CF/PEEK prepreg with a laminate stacking sequence of [0/(0/90)_8_/0]. The fiber volume fractions of the prepreg and the laminate were 57% and 59%, respectively. The laminate had a thickness of 2.4 mm, melting temperature of 330 °C, and initial decomposition temperature of 485 °C. The welding specimens, which were 101.6 mm long and 52.8 mm wide, were cut from the laminate. The cross-section image of the laminate is shown in Figure A1a, which demonstrates good quality, with uniform fiber distribution and without obvious defects. 

The PEEK film was provided by Jilin University Super Engineering Plastics Research Co., Ltd., Jilin, China. Its melting and initial decomposition temperatures were 340 and 537 °C, respectively. The film was 60 μm thick and cut to the dimensions of 200 mm length and 35 mm width for the heating elements.

304 SS (as named by the American Iron and Steel Institute) meshes were provided by Wuxi Tianlong Screen Mesh Co., Ltd., Wuxi, China.

### 2.2. Preparation of Heating Element

**Figure 1:**
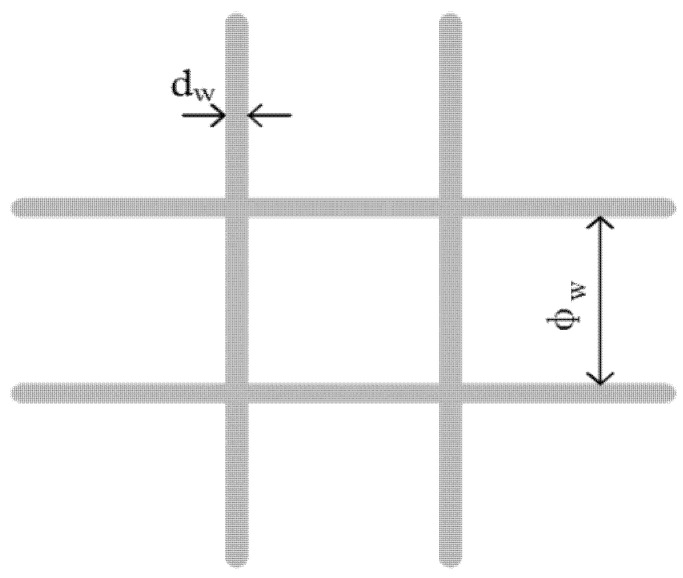
Schematic of stainless steel mesh.

After confirming the ability to perform resistance welding in a preliminary experiment, the three types of plain weave SS meshes from the same manufacturer were chosen as heating elements (Figure 1). The properties of the SS meshes are summarized in Table 1 in which the mesh number (n, mm^−1^) is the number of holes across 25.4 mm of length, and the open gap width (φ_w_, μm) and the open area (F_o_) are defined as

(1) $$\varphi_{w}=\frac{25.4}{n}\times 1000-d_{w}$$

(2) $$F_{o}=\frac{\varphi_{w}{}^{2}}{{\left(\varphi_{w}+d_{w}\right)}^{2}}\times 100\%$$

where $d_{w}$ is diameter of the SS wire in μm [12]. The electrical resistance per unit length (*k*, Ω/m) is the slope obtained by linear fitting the resistance of different lengths of 53 mm-wide metal mesh. In Table 1, mesh number and wire diameter were provided by the producer, and other parameters were calculated.

Equations (1) and (2) imply that the mesh number, open area fraction, and electrical resistance depend on the open gap width and wire diameter. The open gap width and wire diameter therefore determine the resistance welding properties of the SS mesh. 

**Figure 2:**
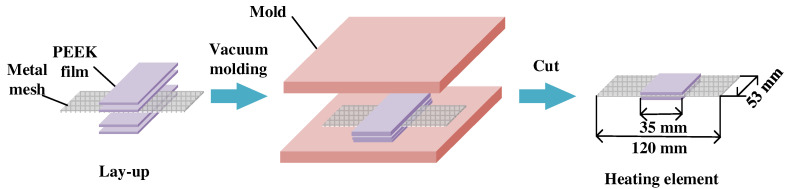
Schematic of preparation method for heating element.

The heating element was fabricated using a vacuum-assisted compression molding process, as shown in Figure 2. The metal mesh was cut to the dimensions of 120 mm length and 53 mm width, and cleaned with acetone in a ultrasonic cleaner for 1 h followed by dry treatment under 60 °C for 0.5 h in an oven (Beijing Zhongleiyuan Technology Development Co., Ltd., Beijing, China). The metal mesh was then sandwiched between two pieces of PEEK films to provide a resin-rich region at the joining interface and to reduce voids due to the entrapment of air in the mesh. The mesh together with the PEEK films was subsequently placed in an aluminum mold and heated to 340 °C under a pressure of 6.5 MPa using a hot press (Qingdao Huabo Machinery Technology Co., Ltd., Qingdao, China). The pressure was maintained under a vacuum of 0.07 MPa for 30 min. Finally, excess portions of the PEEK films were cut out, and a heating element was obtained with the dimensions shown in Figure 2.

### 2.3. Welding Equipment Setup and Welding Process

**Figure 3:**
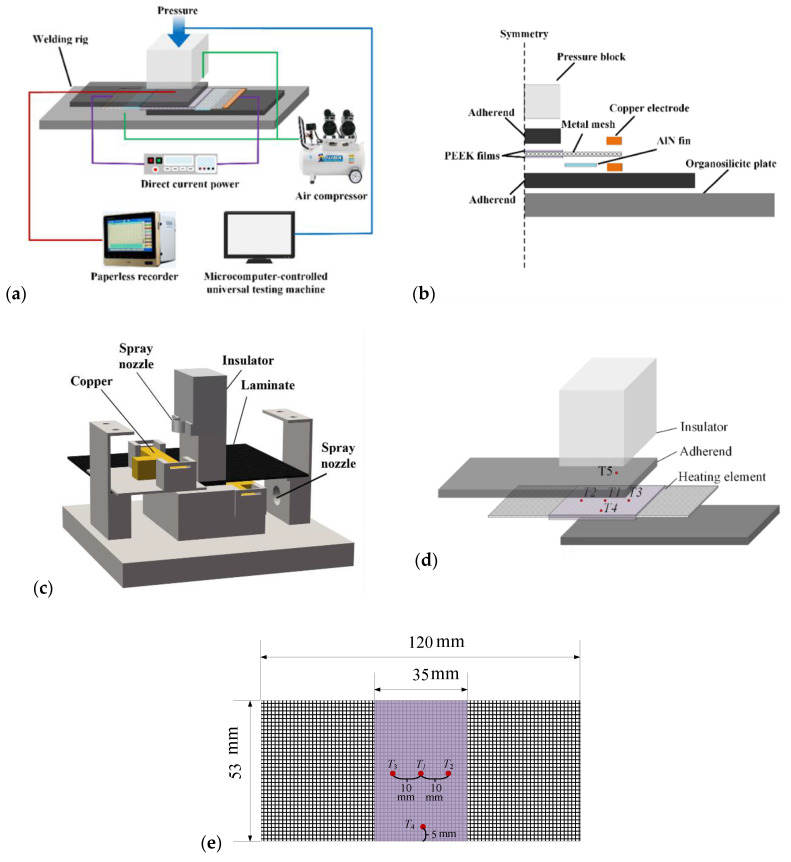
(**a**) Resistance welding system, setup (**b**) section schematic of adherent welding, (**c**) schematic of resistance welding assembly, (**d**,**e**) thermocouple positions.

The resistance welding apparatus consisted of a heating system, monitor system, pressure system, cooling system, and resistance welding rig, as shown in Figure 3a. The heating system supplied direct-current (DC) power at up to 100 A and 20 V through two copper electrodes. The electrodes were joined by bolts and nuts, and both ends of the heating element were clamped under 16.3 MPa of pressure using a torque wrench to reduce contact resistance. The monitoring system consisted of two parts that monitored the temperature and displacement at the welding joint. K-type thermocouples and a paperless recorder were used to monitor the temperature distribution during welding, and a microcomputer-controlled universal testing machine was used to monitor the change in the displacement along the thickness direction of the welded composite. The pressure system consisted of a universal testing machine (ITW group Instron Co., Norwood, MA, USA) and a 53 mm-long and 25.4 mm-wide insulator pressure block. During the welding process, 1.0 MPa pressure was applied to the composite adherend via the insulator pressure block to ensure that the welding adherents were in close contact. The cooling system consisted of an air compressor and aluminum nitride (AlN) fins. The air compressor supplied 0.1 MPa of gas to the edges of the welding area through two spray nozzles to prevent overheating. The AlN fins transferred heat accumulated on the surface of the laminate to the air, which effectively prevented the surface of the laminate from being carbonized. The resistance welding rig was made of an organosilicite material and designed to fix the welded specimen.

**Figure A2:**
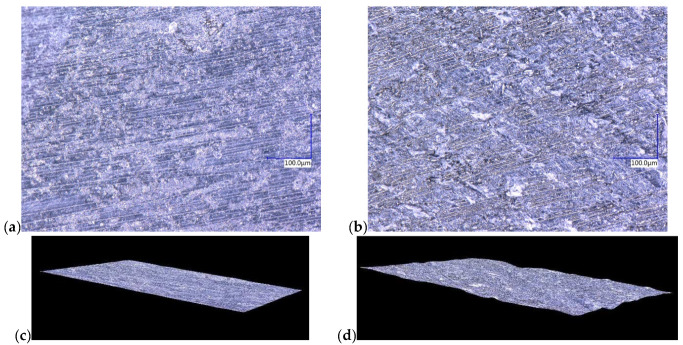
Profilometer image of the composite surface (**a**,**c**) before and (**b**,**d**) after grinding treatment before welding.

Prior to welding, the joining surfaces of the composite adherents were polished using 180 grit silicon carbide sandpaper, and cleaned with acetone followed by dry treatment under 100 °C for 1 h in an oven (Beijing Zhongleiyuan Technology Development Co., Ltd., Beijing, China). The grinding treatment removed the resin layer on the surface and more carbon fibers were exposed, as shown in Figure A2a–d. The surface was smooth before grinding and became rough after grinding, which is beneficial for strong bonding. 

The resistance welding assembly was then installed, as shown in Figure 3b,c. The temperature, displacement, and pressure should be properly controlled to ensure sufficient diffusion of the polymer at the interface and to prevent void formation due to excessive polymer flow out of the joint [7,8]. The heating system determines the heating rate and the temperature distribution at the welding interface. A constant-current DC power output mode was used in this study. To characterize the heating capacity of the heating element, the power density (*µ*, kW/m^2^) defined in Equation (3) is introduced in this study. Note that the power loss due to the imperfect electrical connection of the heating element to the copper electrodes was neglected and the power passing through the heating element was assumed to be equal to that delivered by the electrical source. The resistance of the metal mesh *R_SS_* is given by Equation (4).

(3) $$µ=\frac{P}{LW}=\frac{I^{2}R_{ss}}{LW}$$

(4) $$R_{ss}=kL$$

In Equations (3) and (4), *P* is the heating power due to the Joule heating effect and *L* and *W* are the length and width of the metal mesh, respectively. I is the input current and *k* is the electrical resistance per unit length of the metal mesh with 53 mm width. The power density was adjusted using the alternating current.

The temperature during the welding process was monitored using five K-type thermocouples placed at the positions shown in Figure 3d,e. The thermocouples were coved by insulated tape. Four thermocouples (T_1_–T_4_) were placed between the upper surface of heating element and top adherent at the joining interface. The distance of these thermocouples from the wire mesh was determined by the thickness of the PEEK film in the heating element, which was 60 μm. The first of these four thermocouples was placed at the center of the welding overlap (T_1_), the next two 10 mm apart from the center of the welding overlap (T_2_, T_3_), and the fourth one 5 mm from the edge of the welding overlap (T_4_). A fifth thermocouple was placed at the upper surface center of the top adherent (T_5_). No thermocouples were used in the lap shear test welding samples because the thermocouples could potentially introduce defects into the welding joint and lower the lap shear strength (LSS).

**Figure 4:**
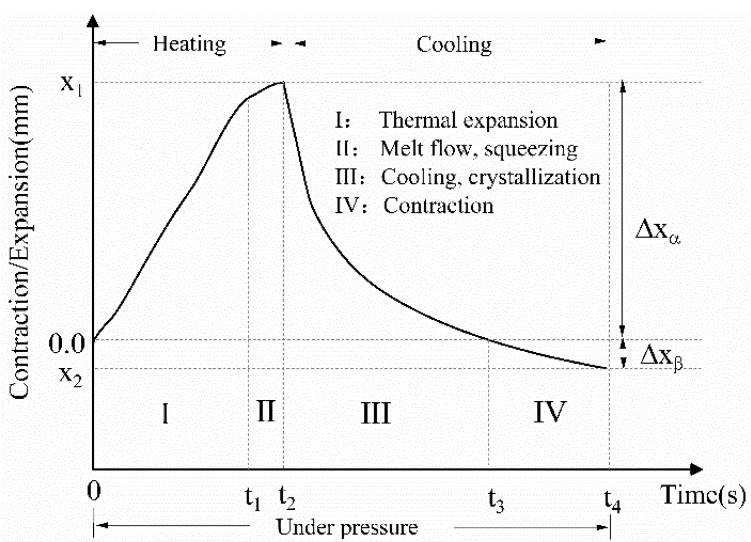
Typical displacement variation in CF/PEEK laminate resistance welding. ΔX_α_: maximum expansion displacement, ΔX_β_: final contraction displacement.

The resistance welding process began after electrical power was turned on and the subsequent application of constant external pressure and gas spraying. The displacement change along the thickness direction of the composite laminate monitored by the universal testing machine is important for understanding the welding process and determining the welding termination and pressure relief points. A typical displacement curve of the lap joint during the welding process is shown in Figure 4. Four distinct stages can be identified in the graph. Stage I is dominated by the volumetric expansion of the composite which led to close contact and an increase in the temperature in the welding joint. In stage II, macroscopic melting flow and squeezing occurred in the welding area and lasted until the power was turned off (welding termination point, t_2_). There was also a decrease in the expansion rate. In stage III, the current was shut off, and the cooling process began. The melt continued to flow until the flow was arrested by the reduction of the transverse pressure or the viscosity increase and crystallization of PEEK. In stage IV, the displacement decreased to below zero. This was mainly caused by the thermal contraction of the lap joint. The pressure was released when the contraction stabilized (pressure relief point, t_4_), and the welding process was completed. ΔX_α_ in Figure 4 indicates the degree of thermal expansion in the lap joint and ΔX_β_ the total deformation of the lap joint after welding.

### 2.4. Characterization

Lap shear tests were conducted to evaluate the welding quality in accordance with ASTM D5868-2014 [24]. After welding, the welded sample was machined to obtain lap shear specimens. The lap shear specimens were 101.6 mm long and 25.4 mm wide and had an overlap width of 25.4 mm and a free length between grips of 72.4 mm. The specimens were tested at a crosshead speed of 13 mm/min using a universal testing machine (Instron 5565, ITW group Instron Co., Glenview, IL, USA). At least five valid tests were performed for each test case to obtain the LSS. After the test, the fractured surfaces were observed using SEM (JSM 7500, JEOL, Tokyo, Japan) to determine the failure mechanism.

## 3. Results and Discussion

### 3.1. Effect of Power Density on Resistance Welding Process

**Figure 5:**
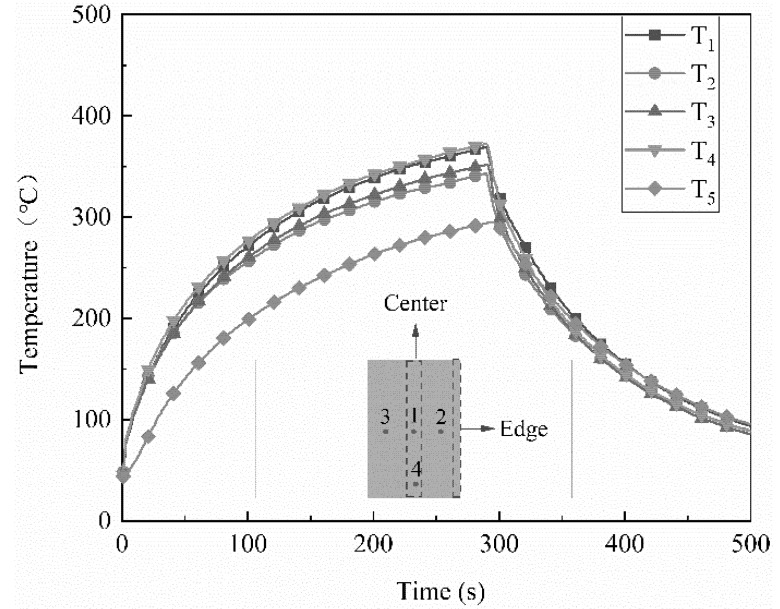
Temperature-time curves of welding specimens obtained with type B heating element at 51 kW/m^2^ power density.

The power density is a key parameter in resistance welding and has a significant effect on the welding process and quality [25]. In this study, different power densities ranging from 51 kW/m^2^ to 82 kW/m^2^ were applied using the type B heating element, the properties of which are listed in Table 1. The corresponding temperature distributions during the welding process were studied. Similar temperature distributions were observed for all the power densities. The temperature distributions at 51 kW/m^2^ are shown in Figure 5. Figure 5 shows that T_1_ was higher than T_2_ and T_3_, indicating that high temperatures occurred at the center of the joining interface and low temperatures at its edges. This is because the edges connected to the metal mesh were exposed to cooling air and heat could be easily released through convection. In addition, T_4_, which was measured close to the edge of the weld overlap without cooling air, was almost equal to T_1_. Therefore, the difference between T_1_ and T_2_ was used to characterize the temperature difference in the welding area. It should be noted that the welding temperature should be higher than the PEEK melting temperature (340 °C) but much lower than the PEEK initial decomposition temperature (485 °C). In the test cases using the type B heating element, the maximum T1 was approximately 420 °C. Moreover, the temperature of the surface of the composite far away from the joining interface should be lower than the melting temperature to avoid obvious deformation of the laminate. To compare the welding effects when the type B heating element was used at different power densities, the welding times at which T_1_ reached 420 °C and T_2_ increased to 340 °C are listed in Table 2, together with the temperature difference at the joining interface when T_2_ reached 340 °C.

It can be seen that the welding time for T_2_ to rise to 340 °C decreased from 279 s to 48 s as the power density increased from 51 kW/m^2^ to 82 kW/m^2^. This corresponds to an increase in the heating rate from 69 °C/min to 400 °C/min. At the same time, the welding time for T_1_ to reach 420 °C also decreased with increasing power density. In particular, the maximum T_1_ was approximately 375 °C and could not reach 420 °C at 51 kW/m^2^ of power density. As the power density increased, T_5_ at the upper surface of the composite decreased from 291 °C to 232 °C when T_2_ reached 340 °C, which is suggestive of a reduction in the heat transfer to the top laminate. This in turn suggests that a high power density produces a high heating rate and an intensively heated region, which is conducive for high welding efficiency. Table 2 also shows that the temperature difference between T_1_ and T_2_ increased from 26 °C to 47 °C as the power density increased from 51 kW/m^2^ to 82 kW/m^2^. This increase in the temperature difference indicates a decrease in the temperature uniformity at the welding area and is attributed to the anisotropic thermal properties of the CF composite. The difference between T_1_ and T_2_ depends on the heat generated by the heating element and heat conduction in the composite. In unidirectional laminates, the thermal conductivity along the length is much higher than that along the width. Ageorges et al. found that the thermal conductivity of CF/PEEK prepreg along the fiber orientation direction and the anisotropy of the thermal conductivity increased significantly with the temperature, which led to a remarkable temperature difference [26]. The larger amount of heat generated at higher power densities led to an increase in the temperature in the welding region, which resulted in a larger thermal property anisotropy of the CF composite. Thus, heat conduction occurred more easily along the length of the composite compared to its width. The larger heat conduction limited the heat accumulation and heating rate at the location of T_2_ and resulted in an increase in the difference between T_1_ and T_2_. When the power density increased to 74 kW/m^2^, the temperature-time curve became sharper, and the heating rate was much higher. This indicates that a significant concentration of heat occurred above 66 kW/m^2^, which is attributed to the interaction between the resistance heat, composite thermal conductivity, and heat dissipation environment. Considering the welding efficiency and heating uniformity, power densities ranging from 58 to 74 kW/m^2^ were chosen for further investigation.

**Figure 6:**
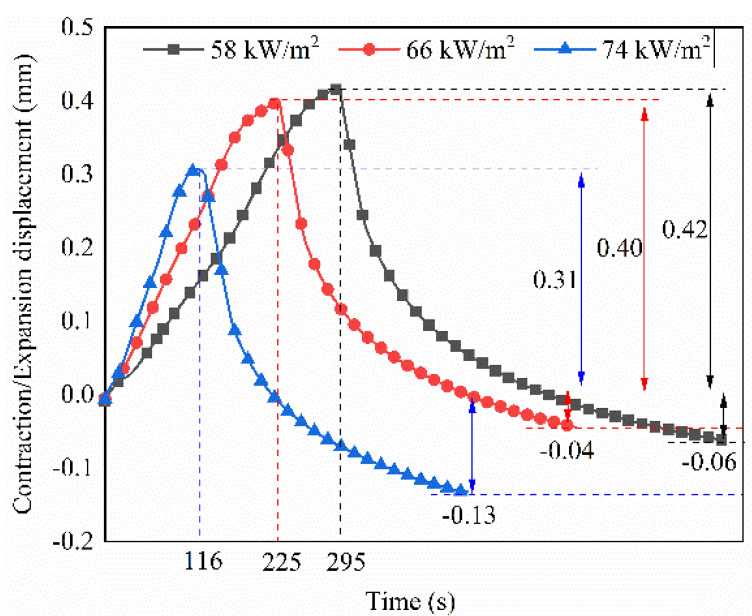
Displacement-time curves of welding specimens obtained with type B heating element at different power densities.

**Figure 7:**
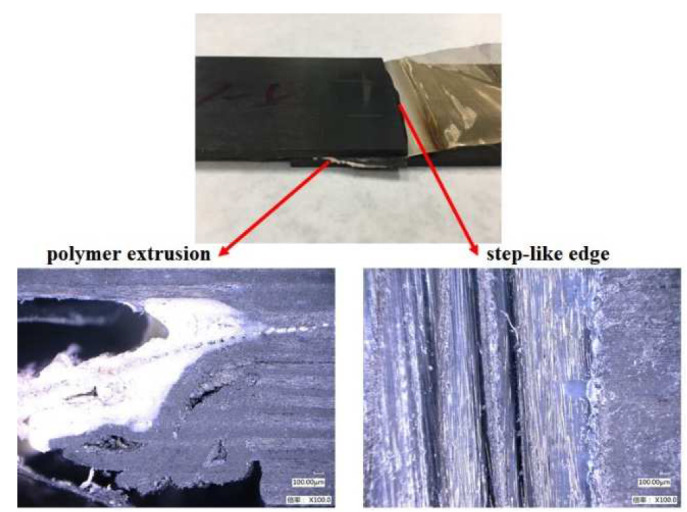
Morphology of weldment obtained with type B heating element at 74 kW/m^2^ power density.

The displacement curves of the weldment using the type B heating element at different power densities are shown in Figure 6. The curves can be divided into four stages, as shown in Figure 4. When the power density increased from 58 kW/m^2^ to 74 kW/m^2^, the lap joint expansion rate in stage I increased. Meanwhile, the maximum expansion displacement ΔX_α_ decreased from 0.42 mm to 0.31 mm. The decrease in ΔX_α_ indicates a reduction in heat transfer to the laminate and therefore, heat concentration in the welded area. The final contraction displacement ΔX_β_ indicates the degree of PEEK and prepreg flow out of the laminate. At 58 kW/m^2^ and 66 kW/m^2^, no visible prepreg slippage occurred and only resin flow occurred, resulting in 0.06 mm and 0.04 mm of ΔX_β_, respectively. However, prepreg slippage was evident at the joining interface at 74 kW/m^2^, at which ΔX_β_ was 0.13 mm. The polymer extrusion and step-like edges of the welded laminate are shown in Figure 7. These features were caused by the large heat accumulation over a short time under a high power density, which resulted in a large deformation of the weldment.

**Figure 8:**
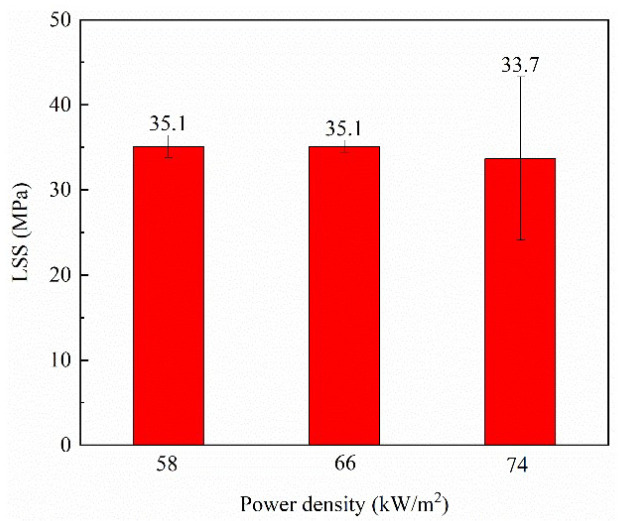
LSS of welded samples obtained with type B heating element at different power densities and welded CF/PEEK composites after surface treatment for SS mesh in [15].

**Figure 9:**
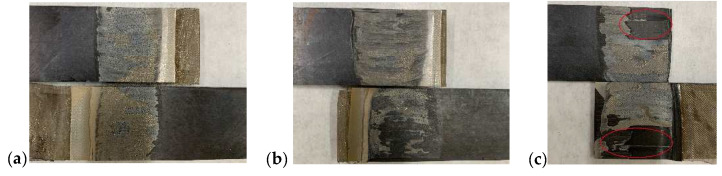
Photos of fracture surfaces of typical welded samples obtained with type B heating element at different power densities of (**a**) 58 kW/m^2^, (**b**) 66 kW/m^2^, (**c**) 74 kW/m^2^. Red circles indicate the positions of laminate damage.

**Figure A3:**
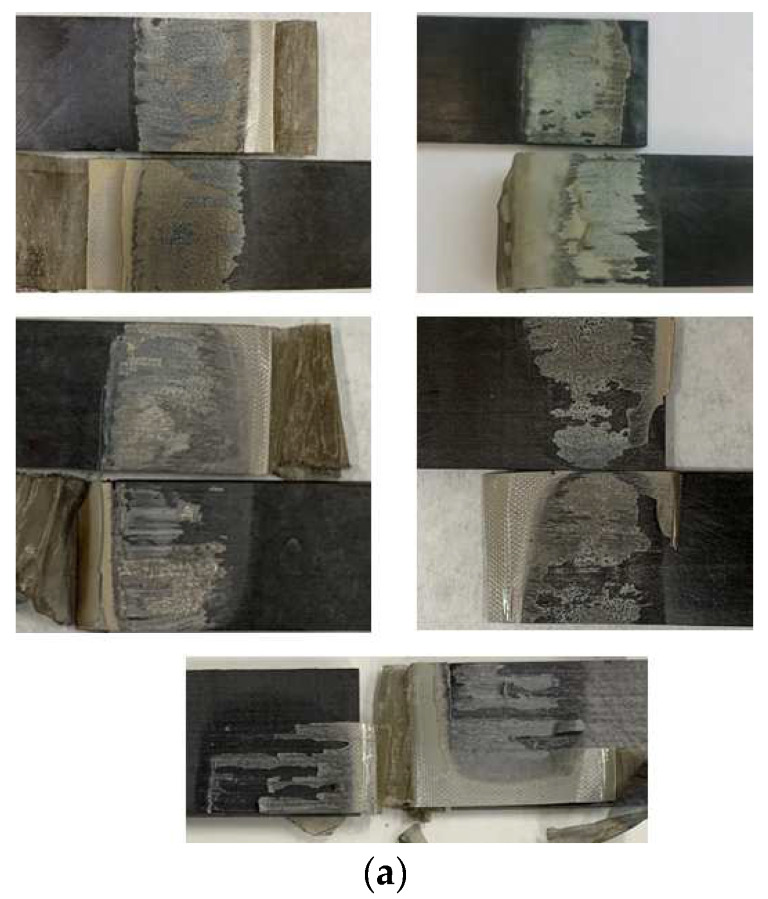

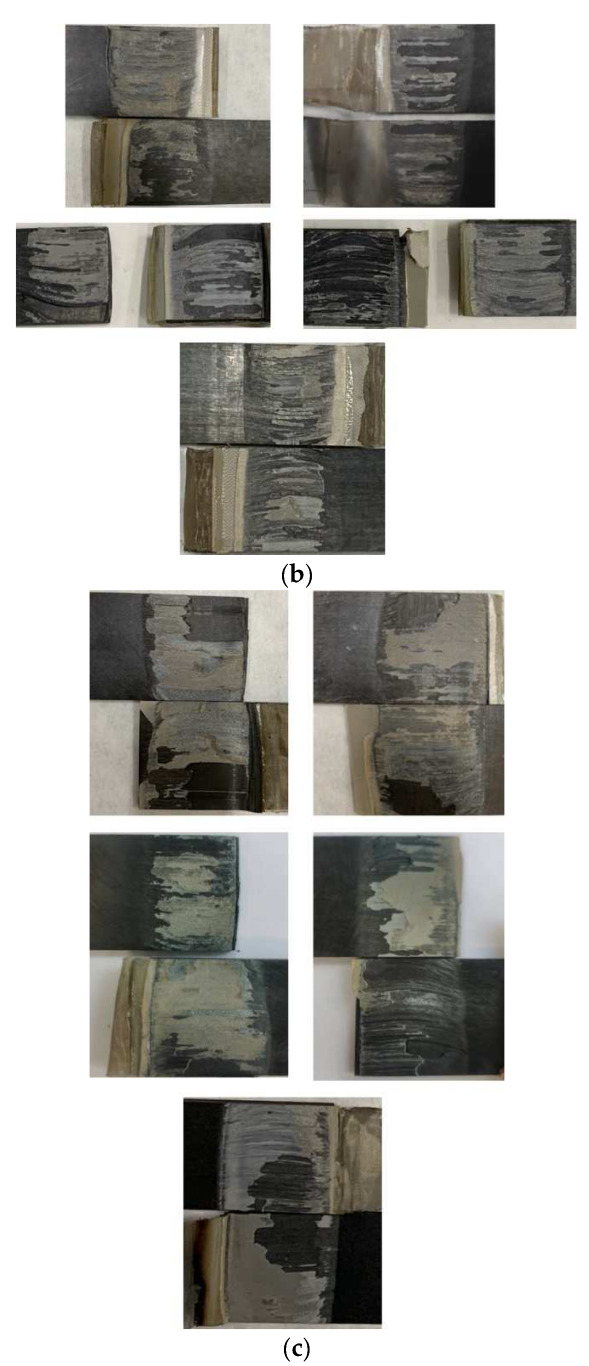
Photos of fracture surfaces of all welded samples obtained with type B heating element at different power densities of (**a**) 58 kW/m^2^, (**b**) 66 kW/m^2^, (**c**) 74 kW/m^2^.

The LSS and photographs of the failure surfaces of the welded specimens obtained using the type B heating element at different power densities are presented in Figure 8 and Figure 9. Photos of fracture surfaces of all welded specimens can be seen in Figure A3. Higher LSS values with acceptable standard deviations were obtained at 58 kW/m^2^ and 66 kW/m^2^. The LSS at 74 kW/m^2^ was slightly lower, and the standard deviation much larger. This shows that the welding quality became unstable when the power density was too high. Figure 9a,b show interlaminar failure of the heating element without laminate damage, which is one of ideal fracture modes [12,27]. The fracture surfaces were covered with a uniform resin layer, and the metal meshes were neatly torn. However, interlaminar failure of the heating element with laminate damage occurred at 74 kW/m^2^, as shown in Figure 9c. This indicates that the bonding strengths of the PEEK film/metal mesh and PEEK film/laminate were higher than the interlaminar strength of the laminate. It is believed that excessive heat accumulation and deformation of the laminate at 74 kW/m^2^ caused obvious thermal stress and defects in the composite, which weakened its mechanical properties and resulted in failure of the composite and large variations in the LSS [28]. Based on the above analysis, 66 kW/m^2^ is the optimized power density that balances welding efficiency with welding quality. In addition, Li et al prepared CF/PEEK weldment using SS heating element and their LSS increased from 28 MPa for SS with sandblasting treatment to 38 MPa for SS with silane grafted treatment [15], as shown in Figure 8. Thus, compared with this work, our results with the optimized power density and SS without chemical treatment are good. The cross-section image of the welded sample is given in Figure A1b, which demonstrates excellent quality with clear joining interface and without defects. 

### 3.2. Effect of Metal Mesh Type on Resistance Welding Process

The three types of SS meshes listed in Table 1 were used to weld CF/PEEK composites. The type A and B metal meshes have the same open area fraction, which is larger than that of the type C metal mesh. The type B and C metal meshes have the same mesh number, whereas the wire diameter of the type C metal mesh is larger. In addition, the resistance of the type B mesh is higher than that of the other meshes. To determine a suitable power density, resistance welding experiments were conducted using the type A and C heating elements at different power densities. The results drawn from the temperature-time curves of the welding specimens are listed in Table 3 and Table 4, respectively. Because of the lower electrical resistance of the type A and C metal meshes, a lower power density was obtained compared with the type B metal mesh for the same input current according to Equation (3). Compared with the results for the type B heating element listed in Table 2, higher welding rates were achieved with slightly larger temperature differences at the joining interfaces using the type A and C heating elements at the same power density. For example, comparing the type B and C heating elements, the heating time at 58 kW/m^2^ decreased from 300 s for the type B element to 101 s for the type C element. Dubé et al. [12] demonstrated that the electrical resistance of the heating element is controlled by the input power and heating rate, and that higher mesh electrical resistances result in lower input powers and longer welding times. The size parameters of the mesh also affect the electrical resistance [12]. These results show that a metal mesh with a lower resistance has a stronger welding capability.

The power densities that resulted in similar welding times and the corresponding temperature differences at the joining interfaces when different types of heating elements were used are listed in Table 5. The power densities required to achieve similar heating rates differed between the different types of heating elements. This might be reason for the differences in the resistivity changes of various metal meshes with increasing temperature after the preparation of the heating element and during the welding process [29]. The temperature gradient at the joining interface when the type A heating element was used was larger than that when the type B and C heating elements were used. Under similar power densities, the temperature distribution was more uneven when the type A heating element was used. It should be noted that the mesh number and open area fraction are determined by the wire diameter and open gap width, as indicated in Equations (1) and (2). Thus, the uneven temperature distribution is attributed to the larger wire diameter and open gap width of the type A heating element.

**Figure 10:**
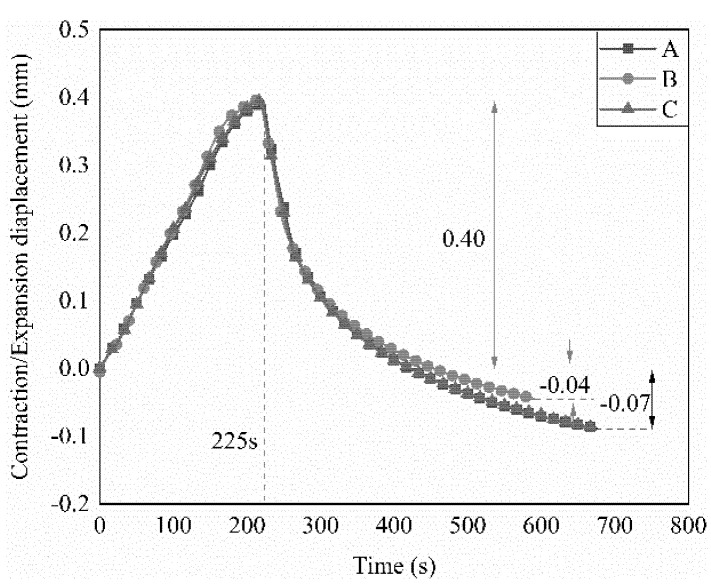
Displacement-time curves of welding specimens obtained with different types of heating elements at the powder densities listed in Table 5.

The displacement curves for the weldments under the conditions listed in Table 5 are shown in Figure 10. The values of ΔX_α_ were almost the same and the values of ΔX_β_ were all less than 0.1 mm. These results indicate that there was appropriate consolidation at the welding regions. The results show that similar welding quality can be obtained using different metal mesh under optimized power density. However, lower power density is obtained for the metal mesh with low electrical resistance under the same input current, and the metal mesh with low resistance has a stronger welding capability at the same power density. Thus, the resistance is the main characteristic of resistance welding for the metal mesh.

**Figure 11:**
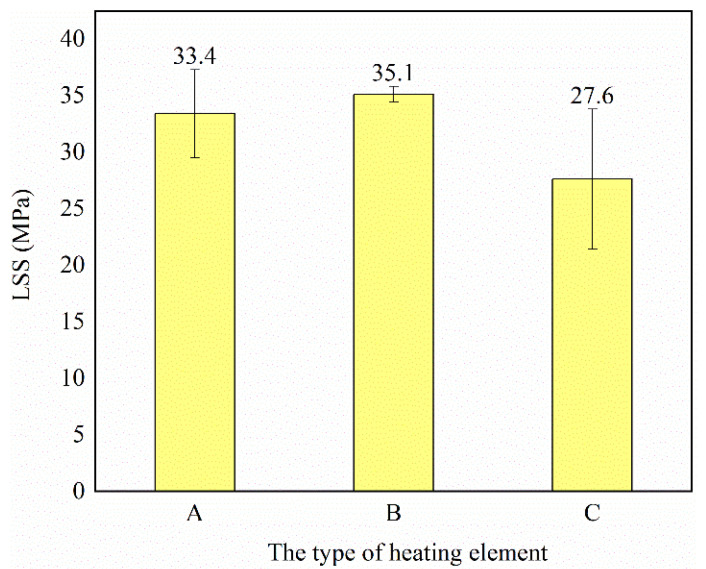
LSS of welded samples obtained with different types of heating elements listed in Table 5.

**Figure 12:**
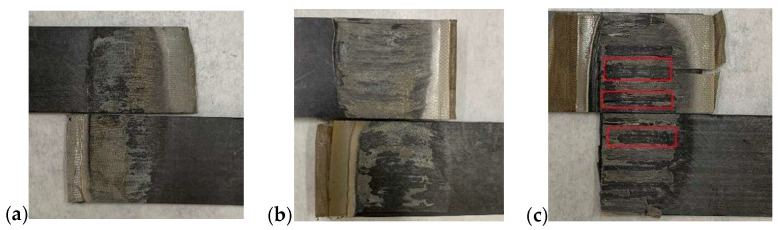
Photos of fracture surfaces of typical welded samples obtained with different heating elements listed in Table 5: (**a**) type A, (**b**) type B, (**c**) type C. Red rectangles indicate the positions of interfacial failure.

**Figure A4:**
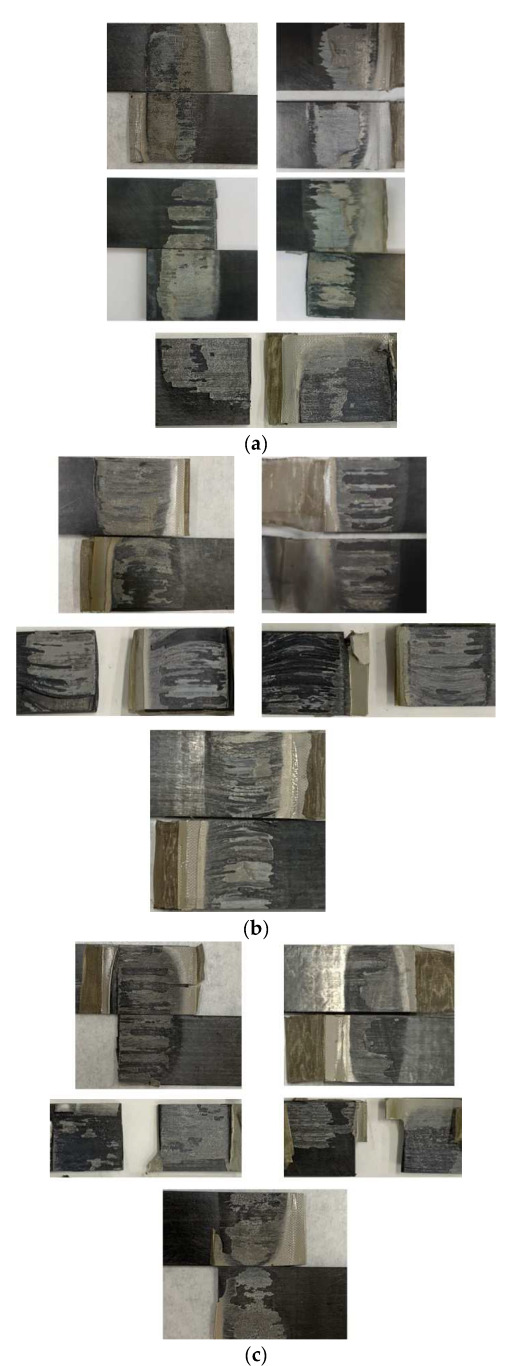
Photos of fracture surfaces of all welded samples obtained with different heating elements listed in Table 5: (**a**) type A, (**b**) type B, (**c**) type C.

The LSS of the samples obtained from Figure 10 were determined and the results are shown in Figure 11. The LSS of the type B heating element was the highest and had the smallest standard deviation, whereas that of the type C heating element was the lowest and had the largest standard deviation. This illustrates that a large open area fraction is necessary to ensure a larger polymer impregnation of the metal mesh and good welding quality. A higher power density and more heat are required to promote polymer diffusion in a mesh with a smaller open area fraction. Moreover, the metal mesh with high ratio of open area fraction and wire diameter is conducive for reinforcing the welding region and strong interface bonding [12], i.e., the type B heating element. It results from smaller inclusion and more polymer sustaining the applied load. The photographs of the failure surfaces of typical welded specimens in Figure 12 demonstrate that the type A and B heating elements lead to interlaminar failure with heating element tearing, while the type C heating element results in interfacial failure with clear debonding between the heating element and the adherend. Photos of fracture surfaces of all welded specimens can be seen in Figure A4a–c. It indicates that the metal meshes bear some part of the applied load, which contributes to high shear strength. 

**Figure 13:**
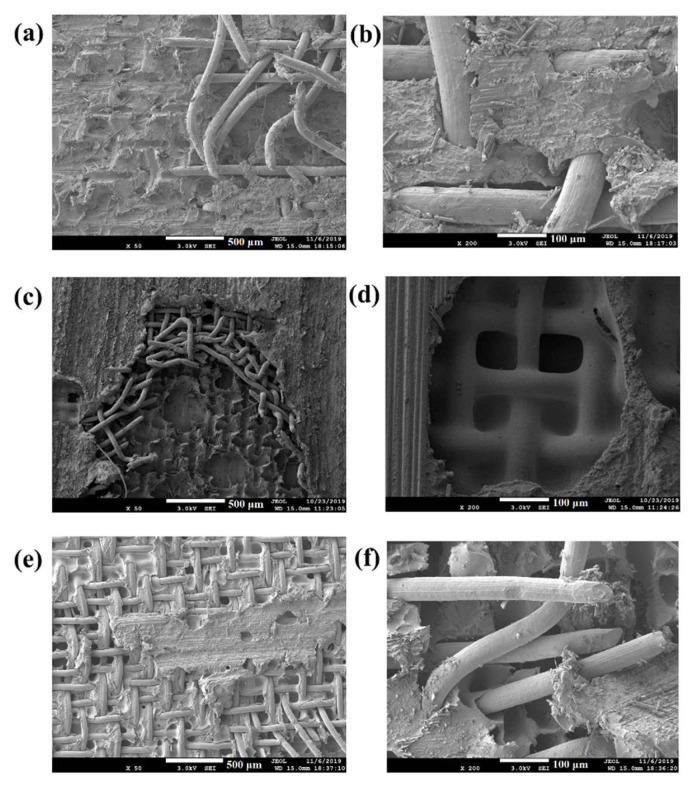
SEM of lap shear failure surfaces with similar LSS obtained with different types of heating elements: (**a**) type A heating element, LSS = 37.3 Mpa, × 50, (**b**) type A heating element, LSS = 37.3 Mpa, × 200, (**c**) type B heating element, LSS = 35.6 Mpa, × 50, (**d**) type B heating element, LSS = 35.6 Mpa, × 200, (**e**) type C heating element, LSS = 34.8 Mpa, × 50, (**f**) type C heating element, LSS = 34.8 Mpa, × 200.

Figure 13 shows the fracture surfaces of adherends with similar values of LSS (approximately 35 MPa) obtained when different types of heating elements were used. The bonding properties are similar to those obtained in CF/PEEK weldment using SS heating elements and an optimized surface treatment method [15]. It can be clearly seen that for all the cases, failure occurred inside the heating elements, followed by the covering of the metal mesh by the polymer, significant deformation of the mesh, and breaking of the metal wire. It is typical interlaminar failure mode with damage in the heating element [4,12]. Figure 13a,b show that significant damage occurred mainly in the type A heating element. The mesh was torn, and the unbroken wires were coated with polymer. Figure 13c,d show that both resin deformation and mesh fracture occurred in the type B heating element. Slight damage on the laminate surface can be seen in Figure 13d, in which the fiber stripes on the mesh are visible. Figure 13e,f show that in the type C element, some wires were broken, but the mesh was much more intact compared to those of the other two heating element types. In addition, there were large parts of composite remaining on the mesh. The damage to the composite adherend indicates good bonding between the composite and the heating element [12]. These results show that mesh and laminate fractures contribute to higher failure loads in the LSS test, and wire with polymer coating has a strong interfacial bonding, which results in large LSS strength. Strong bonding between the metal mesh and polymer, and sufficient loading on the metal mesh under suitable power density are conducive for high welding quality.

## 4. Conclusions

An experimental investigation on the resistance welding of CF/PEEK laminates using various SS heating elements at different power densities ranging from 29 to 82 kW/m^2^ was conducted in this study. A resistance welding system was established, and a typical four-stage welding process performed. The effects of the power density and SS mesh geometry on the welding process and bonding properties of the joint were studied. The conclusions are summarized as follows:

As the power density increased from 51 kW/m2 to 82 kW/m^2^, the heating rate increased from 69 °C/min to 400 °C/min, and the temperature distribution became more uneven at the joining interface, at which the maximum temperature was close to 50 °C. At low power densities, the heating rate is low because of excessive heat loss and a long welding time is required to melt the matrix at the joining interface. A higher power density leads to a larger heat concentration on the welded area, resulting in a faster welding rate and an increase in the laminate deformation (more than 0.1 mm contraction along the thickness) due to prepreg slippage and polymer extrusion. Excessive heat accumulation and composite deformation lead to damage in the composite far away from the joining interface.

Three types of SS meshes with different mesh numbers, wire diameters, open gap widths, open area fractions, and electrical resistivities were investigated in this study. A large wire diameter and open gap width, which resulted in low resistance in the type A heating element, can easily lead to an uneven temperature distribution. However, the low resistance results in a short welding time under a given power density. Compared with the type B mesh, which has a high resistance, and similar welding times and consolidation degrees were obtained at low power densities for the type A and C meshes, both of which have low resistance. 

The LSS results demonstrate that a large open area fraction and a small wire diameter in the metal mesh are conducive for strong interfacial bonding because of sufficient polymer impregnation into the metal mesh and small inclusion in the welding region. An LSS of approximately 35 Mpa can be obtained with an appropriate power density for all the mesh types. High shear strength is followed by interlaminar failure mode with damage in the heating element or the adherends. Strong bonding between the metal mesh and polymer, and sufficient loading on the metal mesh under suitable power density are conducive for high welding quality.

## Figures and Tables

**Table 1:** Properties of stainless steel meshes.

Type of Stainless Steel Mesh	A	B	C
Mesh number, n (mm^−1^)	100	200	200
Wire diameter, d_w_ (μm)	80	40	50
Open gap width, φ_w_ (μm)	174	87	77
Open area fraction, F_o_ (%)	46.9	46.9	36.8
Electrical resistance per unit length, *k* (Ω/m)	0.868	1.976	0.942

**Table 2:** Welding time at specific temperatures and temperature differences at the joining interface obtained with type B heating element at different power densities.

Power Density /(kW/m^2^)	Time When T_2_ Reached 340 °C/(s)	Time When T_1_ Reached 420 °C/(s)	Temperature of T_5_ When T_2_ Reached 340 °C/(°C)	Difference between T_1_ and T_2_ When T_2_ Reached 340 °C/(°C)
51	279	/	291	26
58	201	300	285	33
66	140	223	271	32
74	55	74	244	44
82	48	60	232	47

**Table 3:** Welding time at specific temperatures and temperature differences at the joining interface obtained with type A heating element at different power densities.

Power Density /(kW/m^2^)	Time When T_2_ Reached 340 °C/(s)	Time When T_1_ Reached 420 °C/(s)	Difference between T_1_ and T_2_ When T_2_ Reached 340 °C/(°C)
29	/	/	/
36	/	/	/
44	241	320	44
49	160	222	36
53	121	165	35

**Table 4:** Welding time at specific temperatures and temperature differences at the joining interface obtained with type C heating element at different power densities.

Power Density /(kW/m^2^)	Time When T_2_ Reached 340 °C/(s)	Time When T_1_ Reached 420 °C/(s)	Difference between T_1_ and T_2_ When T_2_ Reached 340 °C/(°C)
31	/	/	/
44	156	230	32
45	139	202	30
48	114	162	29
53	91	124	32
58	78	101	35

**Table 5:** Power density, welding time at specific temperatures and temperature differences at the joining interface obtained with different types of heating elements at similar heating rates.

Type of Heating Element	Power Density/ (kW/m^2^)	Time When T_2_ Reached 340 °C/(s)	Time When T_1_ Reached 420 °C/(s)	Difference between T_1_ and T_2_ When T_2_ Reaches 340 °C/(°C)
A	49	160	222	36
B	66	140	223	32
C	44	156	230	32
